# Coronary Artery Ectasia Presenting as ST-Elevation Myocardial Infarction: An Intravascular Ultrasound-Guided Percutaneous Coronary Intervention Strategy and Case-Based Review

**DOI:** 10.31083/RCM46098

**Published:** 2026-03-17

**Authors:** Qianfeng Xiong, Shaoyong Chen, Wenbo Li, Yaowu Xie

**Affiliations:** ^1^Department of Cardiology, Fengcheng People’s Hospital, The Affiliated Fengcheng Hospital of Yichun University, 331100 Fengcheng, Jiangxi, China; ^2^Department of Cardiology, Jiangxi Provincial People’s Hospital, The First Affiliated Hospital of Nanchang Medical College, 330000 Nanchang, Jiangxi, China

**Keywords:** coronary aneurysm, ST elevation myocardial infarction, ultrasonography, interventional, percutaneous coronary intervention, anticoagulants, drug-eluting stents

## Abstract

Coronary artery ectasia (CAE) is characterized by abnormal, localized, or diffuse dilatation of the coronary vasculature and is an increasingly recognized anatomical entity encountered during coronary angiography. Although often associated with atherosclerosis, the exact pathogenesis of CAE remains unknown; hence, an optimal management strategy is difficult to establish and remains highly controversial due to a lack of high-quality randomized controlled trial evidence. Current therapeutic modalities include medical therapy, percutaneous coronary intervention (PCI), and surgical options. We present a review, supported by a representative case of ST-elevation myocardial infarction (STEMI) in a patient with CAE, as a systematic summary of the clinical and angiographic features of the condition. We discuss contemporary treatment approaches, especially how to navigate antithrombotic strategies and the role of intravascular ultrasound (IVUS)-guided PCI.

## 1. Introduction

Coronary artery ectasia (CAE) is an anatomical abnormality characterized by the focal or diffuse dilatation of the epicardial coronary arteries, conventionally defined as a diameter ≥1.5 times that of an adjacent reference segment [1]. The prevalence of CAE has been reported to be highly variable due in large measure to the lack of consistency in the diagnostic definition used in a variety of studies [2].

The precise pathogenesis of CAE remains unexplained. Though atherosclerosis is espoused as the major cause in the majority of the patients, systemic inflammatory vasculopathies, connective tissue disorders, and congenital insults have all been related to its development [3]. Clinical manifestations are very variable, from asymptomatic presentations, wherein CAE is usually an incidental finding, to symptomatic states presenting as angina or acute coronary syndrome (ACS) [4].

A lack of rigorous comparative evidence has prevented the definition of a uniform management strategy. Therapeutic choices usually depend on clinician experience and local customs, extending from medical therapy, including risk-factor control and antithrombotic strategies, to percutaneous exclusion of aneurysmal segments and surgical options.

This case-anchored narrative review attempts to accomplish the following: (1) present a typical case of ST-elevation myocardial infarction (STEMI) in the context of CAE, with a focus on interventional challenges related to the ectatic, thrombus-laden culprit segment; (2) systematically review recent evidence on definition, epidemiology, pathogenesis, clinical presentation, and management of ectatic coronary disease, mainly focusing on antithrombotic strategies; and (3) provide an intravascular imaging-based practical framework for management of ectatic culprit vessels.

## 2. Scope and Methods of the Review

We searched PubMed/MEDLINE and Embase from inception to August 2025 using combinations of “coronary artery ectasia”, “coronary artery aneurysm”, “coronary aneurysm”, “STEMI”, “ACS”, “myocardial infarction”, “no-reflow”, “slow flow”, “intravascular imaging”, “percutaneous coronary intervention (PCI)”, “deferred stenting”, “anticoagulation”, and “antithrombotic therapy”.

We included observational cohorts, randomized or quasi-randomized trials, and case series reporting definitions, epidemiology, pathophysiology, clinical presentation, antithrombotic therapy, or revascularization outcomes. Single-patient case reports were used selectively to illustrate technical nuances. Non–peer-reviewed items, conference abstracts, and non-human studies were excluded. Two reviewers independently screened and assessed full texts; disagreements were resolved by consensus. Risk of bias was appraised based on study design, confounding control, and outcome adjudication.

Records identified: PubMed 240 and Embase 678; after de-duplication, 741 remained; 291 full texts were assessed; 38 studies were included in the qualitative synthesis, consisting of 2 randomized controlled trials, 4 systematic reviews, 25 cohort studies, and 7 case reports. Given heterogeneity and the narrative objective, no quantitative pooling was undertaken.

## 3. Case Vignette: CAE Presenting as STEMI

**Fig. 1.**
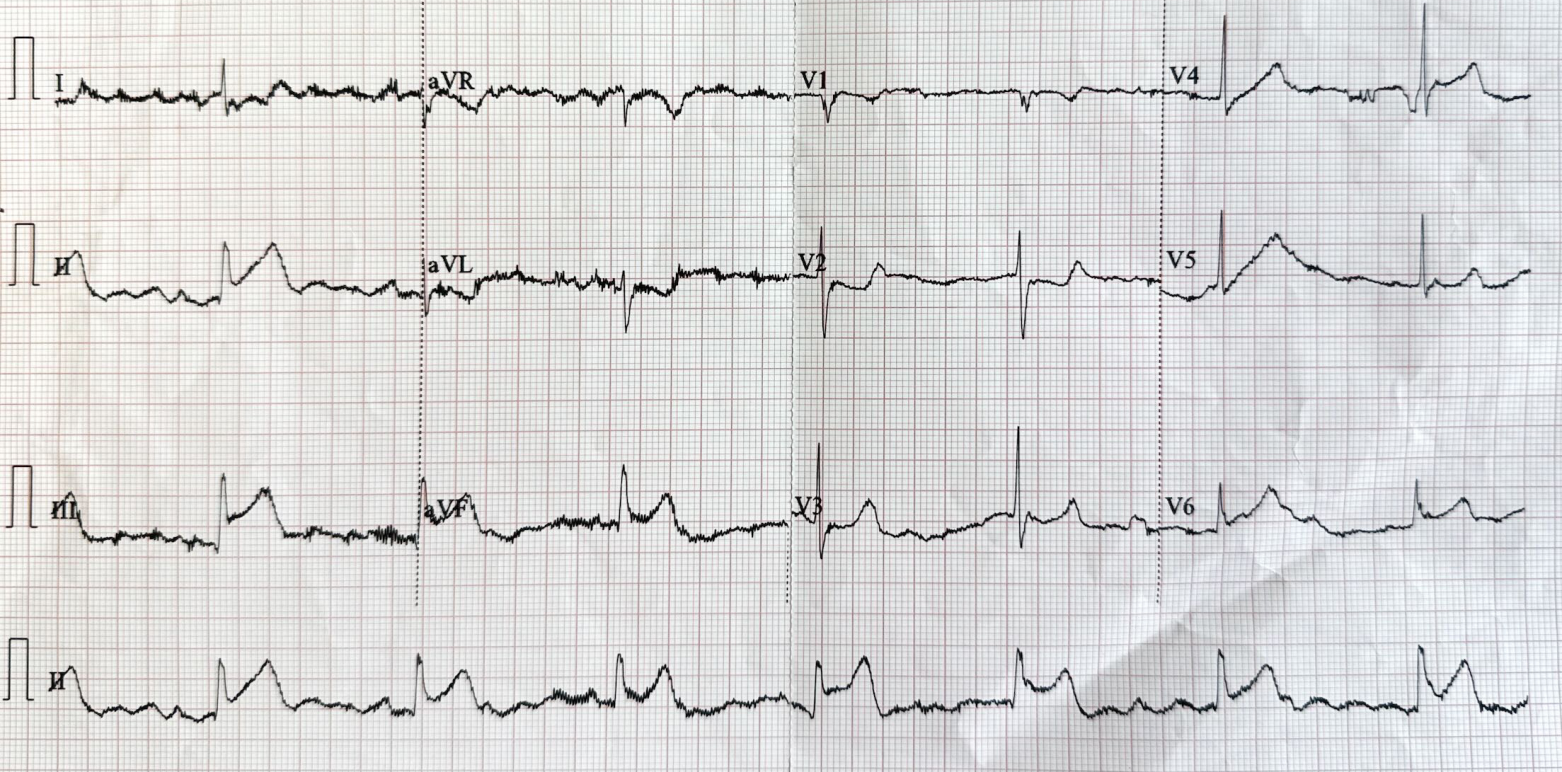
**Twelve-lead electrocardiogram (25 mm/s, 10 mm/mV)**.

A 79-year-old woman presented with 2 hours of persistent, crushing chest pain accompanied by a sense of impending doom, pallor, and diaphoresis. On arrival, she was hemodynamically stable with a blood pressure of 112/79 mmHg and a heart rate of 44 bpm. Her medical history included obesity, Parkinson’s disease, and type 2 diabetes mellitus without regular glucose-lowering therapy. The initial 12-lead electrocardiogram showed sinus bradycardia and ST-segment elevation >0.2 mV in the inferior leads (II, III, aVF), consistent with inferior-wall STEMI (Fig. 1).

**Fig. 2.**
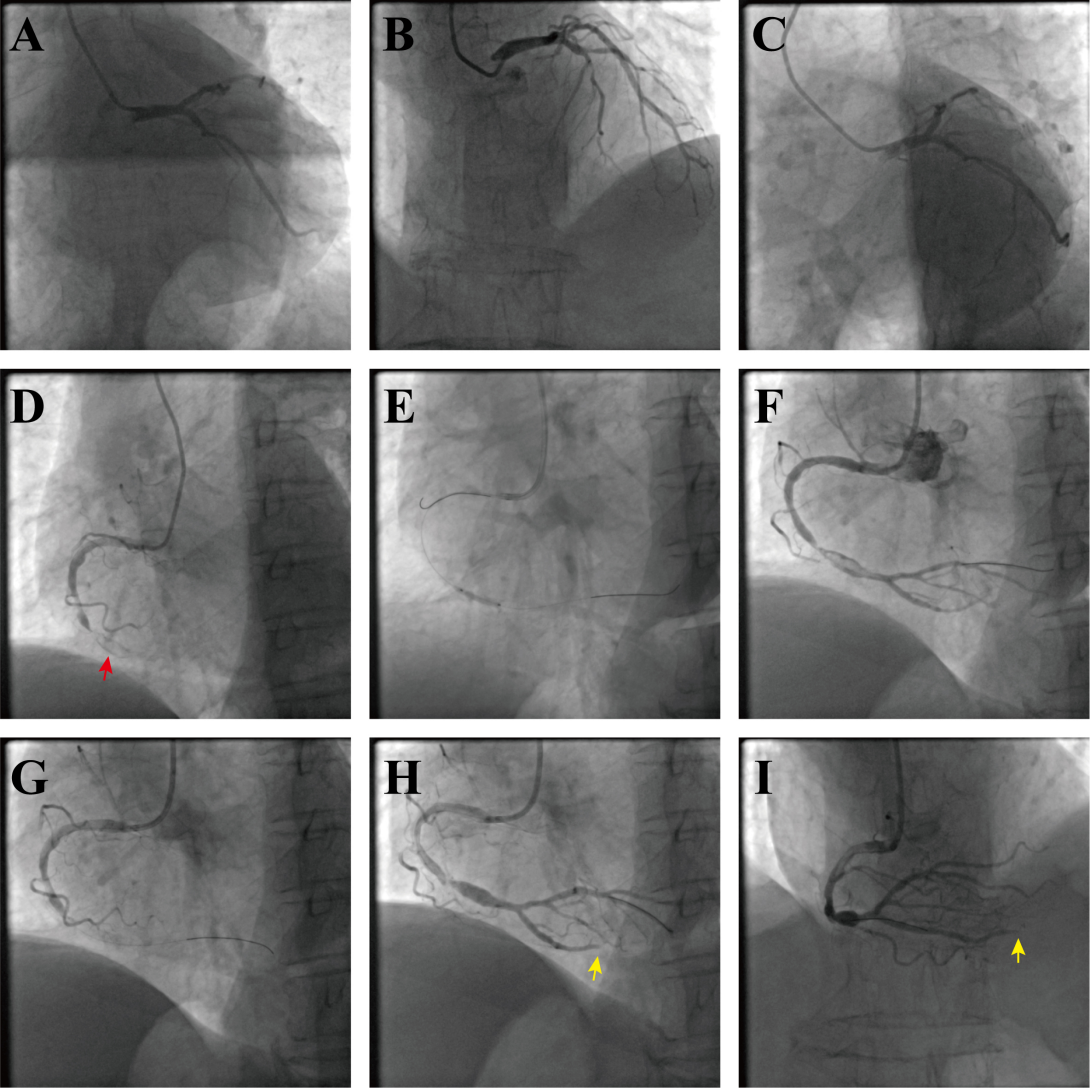
**Coronary angiography and procedural course**. (A) Caudal projection of the left coronary system. (B) Cranial projection demonstrating diffuse stenosis of the left anterior descending artery (LAD). (C) “Spider” view of the left system. (D) Left Anterior Oblique (LAO) 45° view showing total occlusion of the mid right coronary artery (RCA); the red arrow highlights an ectatic segment with superimposed thrombus. (E) After guidewire crossing, balloon predilation is performed. (F) Angiography shows restoration of antegrade flow. (G) Following manual aspiration thrombectomy, angiography demonstrates no-reflow. (H) After intracoronary nitroprusside and tirofiban, flow improves; the yellow arrow indicates thrombotic occlusion of the distal posterior descending artery (PDA). (I) Cranial projection again shows persistent distal PDA thrombotic occlusion (yellow arrow).

Coronary angiography demonstrated diffuse stenoses in the left anterior descending (LAD) and left circumflex (LCx) arteries (Fig. 2A–C), as well as total occlusion of the mid right coronary artery (RCA) with marked ectatic dilation and a heavy thrombus burden (Fig. 2D). A guidewire was advanced across the RCA occlusion, and antegrade flow was restored after balloon predilation (Fig. 2E,F). Manual aspiration failed, and subsequent angiography revealed a no-reflow phenomenon (Fig. 2G). Intracoronary tirofiban and sodium nitroprusside were administered, resulting in reperfusion; however, thrombotic occlusion persisted in the distal posterior descending artery (PDA) (Fig. 2H,I). As the patient’s chest pain had substantially improved, a staged strategy was adopted with continued systemic heparin anticoagulation and a planned re-look angiogram.

**Fig. 3.**
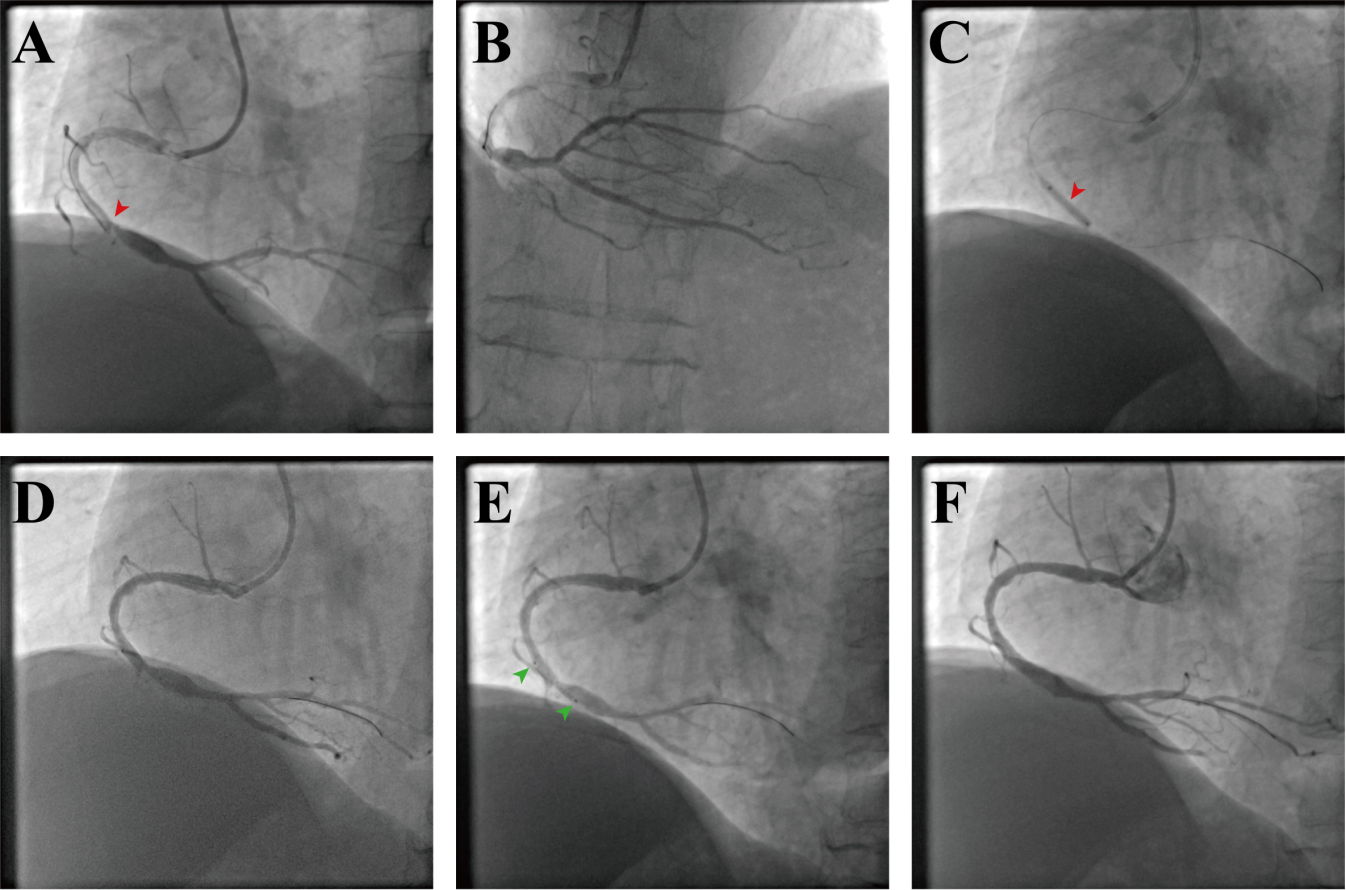
**Staged right coronary PCI and final result**. (A) LAO 45° projection at re-look angiography demonstrating Thrombolysis in Myocardial Infarction (TIMI) grade 3 flow in the right coronary artery (RCA); the red arrow points to the stenosis. (B) Cranial projection with opacification of the distal posterior descending artery (PDA). (C) Balloon predilation of the focal stenosis (red arrow). (D) Post-predilation angiogram showing lesion relief; an IVUS catheter is advanced for sizing and landing-zone assessment. (E) Stent positioning across the culprit segment (4.0 × 13 mm drug-eluting stent; two green arrows point to the stent markers). (F) Final angiogram after post-dilation with noncompliant balloons (up to 4.5 mm) showing optimal expansion/apposition on IVUS and brisk TIMI 3 flow without residual stenosis or dissection.

**Fig. 4.**
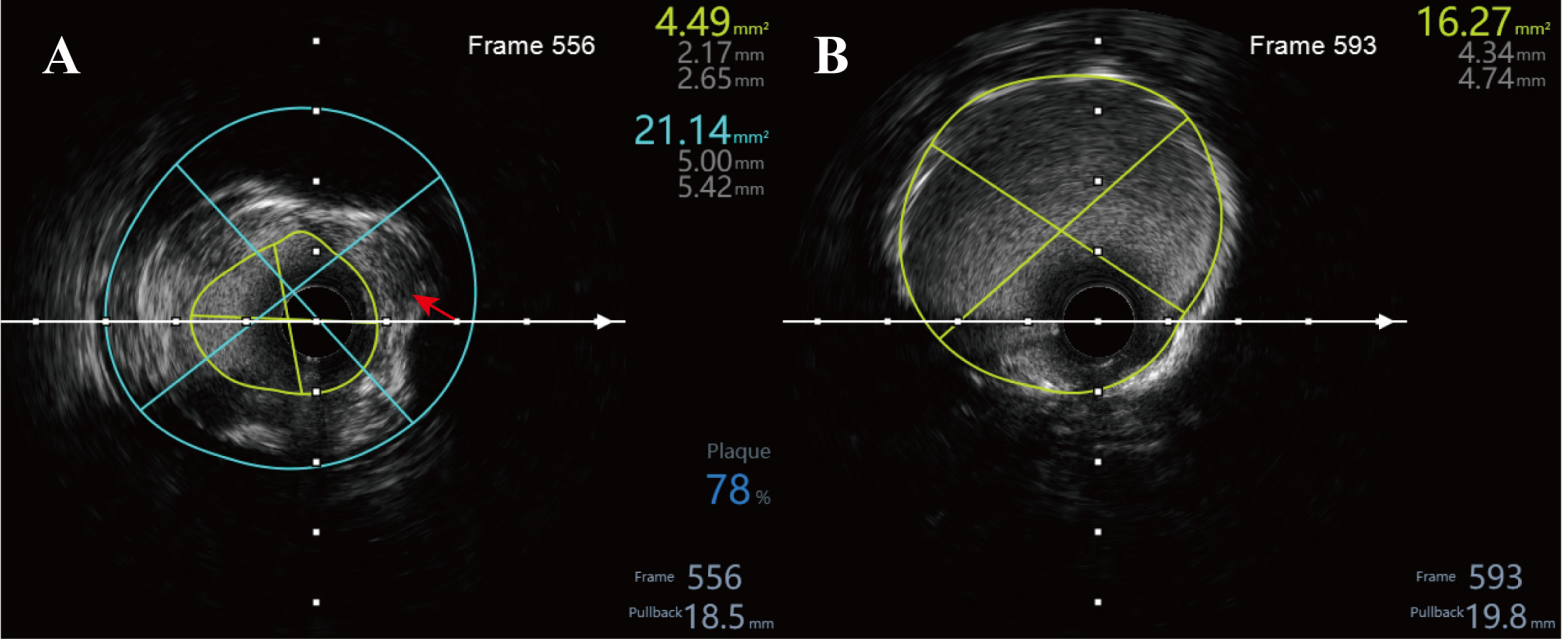
**Intravascular ultrasound (IVUS) assessment**. (A) After balloon predilation at the culprit right coronary artery (RCA) stenosis, IVUS shows a minimal lumen area (MLA) of 4.49 mm^2^ (minimum/maximum lumen diameters 2.17/2.65 mm) and an external elastic membrane (EEM) area of 21.14 mm^2^, corresponding to an estimated plaque burden ≈78%; the red arrow indicates a coronary dissection with intramural hematoma. (B) Following drug-eluting stent implantation and optimization, IVUS demonstrates a minimal stent area (MSA) of 16.27 mm^2^ (minimum/maximum stent diameters 4.34/4.74 mm) with good expansion and apposition and no edge dissection.

On the control angiography, the thrombus burden in the RCA had nearly resolved, and the distal PDA was recanalized with Thrombolysis in Myocardial Infarction (TIMI) grade 3 flow (Fig. 3A,B). A severe stenosis proximal to the ectatic segment remained. Given the high risk of re-occlusion, intravascular ultrasound (IVUS) was performed after balloon predilation (Fig. 3C,D, and Fig. 4A). A drug-eluting stent was deployed across the culprit lesion with a slight overlap into the ectatic portion (Fig. 3E), followed by post-dilation using noncompliant balloons. Repeat IVUS confirmed optimal expansion and apposition (Fig. 3F and Fig. 4B). The planned antithrombotic regimen was rivaroxaban (10 mg tablets, Bayer AG, Leverkusen, North Rhine-Westphalia, Germany) added to dual antiplatelet therapy (aspirin [100 mg tablets, Bayer AG, Leverkusen, North Rhine-Westphalia, Germany] and clopidoggrel [75 mg tablets, Sanofi, Paris, Île-de-France, France]). The patient was asymptomatic at the 1-month follow-up, with a repeat coronary angiography scheduled for 6–12 months.

## 4. Definitions and Epidemiology: Untangling Ectasia vs Aneurysm

A clear consensus on the nosology of coronary artery dilatation is still lacking. This inconsistency in terminology has significantly hampered the synthesis of available evidence. To be precise, the definitions distinguish between focal and diffuse forms: a coronary artery aneurysm (CAA) is a localized enlargement (≥1.5 times the adjacent reference diameter) involving less than 50% of the vessel’s length. In contrast, CAE is defined as a diffuse dilatation involving 50% or more of the vessel length [1, 2, 3]. The most commonly used topographical scheme for anatomic description is the Markis classification [5]: Type I, diffuse ectasia in two or three vessels; Type II, diffuse ectasia in one vessel plus localized ectasia in another; Type III, diffuse ectasia confined to a single vessel; and Type IV, localized/segmental ectasia. The differentiation between CAE and CAA is not merely semantic, as it changes treatment strategies and influences prognosis. Observational data indeed suggest that diffuse/multi-vessel ectasia (Markis class I–II) carries a higher risk of ACS and major adverse cardiovascular events (MACE) than do localized lesions, possibly due to prothrombotic hemodynamic features [5, 6]. These findings advocate phenotype-guided therapy and emphasize the need for prospective imaging-guided studies.

Reported prevalence among patients undergoing coronary angiography ranges from 0.3% to 5% [1, 2]. This wide range reflects heterogeneity in definitions, imaging thresholds, and study populations. Studies that count only CAA naturally yield lower estimates than those that pool CAA and CAE [1, 7, 8]. True frequency may be underestimated because diffuse disease often lacks a clear reference segment, making the 1.5-fold criterion difficult to apply [9]. Conversely, angiography-based cohorts may overestimate prevalence relative to the general population because catheterization is undertaken in selected, symptomatic individuals [10].

Risk factors overlap partially with those for atherosclerotic coronary disease. Male sex, cigarette smoking, hypertension, and dyslipidemia are consistently associated with coronary dilatation [11, 12, 13, 14]. Intriguingly, diabetes mellitus shows an inverse association with CAE in several series [15]. Proposed mechanisms include enhanced extracellular matrix glycation and accumulation of advanced glycation end products, which increase vascular stiffness and may limit outward remodeling [16, 17], as well as diabetes-related negative remodeling that impairs compensatory enlargement of the vessel wall [18, 19]. CAE may coexist with peripheral arterial disease, abdominal aortic aneurysm, and valvular anomalies [20]. Proximal coronary segments are more frequently involved than distal segments; the RCA is most often affected, followed by the left anterior descending and the circumflex, whereas left main involvement is uncommon [19]. Hemodynamic and geometric factors—higher pulsatile pressure and shear stress near the coronary origins, vessel curvature, branching, and turbulence in the RCA—are plausible contributors to this distribution. Although an inverse association between diabetes and CAE has been documented, the patient in our report, an elderly woman with diabetes, developed an ectatic lesion in the RCA.

## 5. Etiology and Pathophysiology: Atherosclerosis, Inflammation, and Remodeling

The exact cause of CAE is still incompletely understood. While a genetic predisposition has been suggested [21], in adults, the disorder is considered a result of an atherosclerotic process and contributes to more than half of the cases [3]. An atherosclerotic hypothesis is thereby supported by shared risk factors with typical coronary artery disease and also by similar histopathological findings, such as an accumulation of lipids, hyalinization, and disruption of the elastic fibers in the arterial wall [22]. Despite these similarities, CAE also exhibits features uncharacteristic of ordinary plaques, including a relatively preserved intima with a loss of medial elastic components. These features are indeed thought to be central in the ectatic remodeling process [23].

An inflammatory milieu probably exacerbates this process. This hypothesis is supported by data linking the extent of coronary dilatation to systemic concentrations of specific mediators. For example, circulating levels of soluble adhesion molecules (intercellular adhesion molecule-1, vascular cell adhesion molecule-1, and E-selectin), monocyte chemoattractant protein-1, and C-reactive protein all show a correlation with the severity of the condition [24, 25, 26]. Histopathologic studies describe diffuse vascular inflammation, with up-regulation of matrix metalloproteinases (MMPs) that degrade connective-tissue proteins, thereby weakening the vessel wall [27]. Taken together, these observations suggest that although CAE intersects with atherosclerosis, it is not simply a variant of occlusive coronary disease; in many patients, it may represent a systemic vasculopathic remodeling phenotype expressed in the coronary circulation [28].

Beyond atherosclerosis, inflammatory and connective-tissue disorders contribute importantly to CAE, including Marfan syndrome, Kawasaki disease, and systemic lupus erythematosus [29, 30, 31]. Kawasaki disease is the leading cause of coronary dilatation in children; in Japanese cohorts, approximately 20% of affected children develop coronary ectasia/aneurysm in some series [32]. Enhanced MMPs activity has also been documented in Kawasaki disease [33], providing a mechanistic link to extracellular-matrix degradation similar to that proposed in adult CAE.

Another pathway includes iatrogenic lesions. In such cases, during PCI, stent oversizing, or high-pressure balloon inflation, injury to the arterial wall could result in dissection with subsequent aneurysmal or ectatic remodeling as healing occurs [34, 35]. It is also important to note that congenital and iatrogenic lesions tend to be single-vessel lesions, while those of atherosclerotic and vasculitic origin usually involve multiple coronary arteries [34]. These various etiologies again underscore the importance of individual patient-oriented diagnostic workup and management approaches.

## 6. Clinical Presentation: Spectrum From Silent to ACSs

The majority of individuals with CAE remain asymptomatic, with the diagnosis being usually made incidentally during coronary angiography or coronary CT. For the subset of patients who are symptomatic, there is a heterogeneous range of clinical presentations, including:

(1) Angina pectoris. About 50% report episodes of angina of variable duration [36]. Proposed mechanisms include distal hypoperfusion, microembolization, and slow or turbulent flow within ectatic segments.

(2) ACSs. A significant proportion of patients with CAE present directly with ACS, and the incidence of ACS is higher in Markis types I-II than in types III-IV [5]. A retrospective analysis has shown that the proportion of CAE patients presenting with ACS reached 54%, with STEMI patients accounting for over 40% of this group [37]. The ectatic vascular segments exhibit abnormal hemodynamic characteristics, including prolonged stasis, increased turbulence, slow flow, and reduced shear stress, all of which are high-risk factors that promote *in situ* thrombosis and distal embolization. These characteristics can trigger or exacerbate STEMI events, independent of classic plaque rupture. In STEMI associated with CAE, the culprit vessel itself is often ectatic, which is consistent with the theory of local thrombus formation within the dilated segment [38].

(3) Cardiac tamponade/cardiogenic shock. Although rare, rupture-related tamponade and hemodynamic collapse are catastrophic complications that require urgent recognition and intervention [39].

(4) Non-chest-pain symptoms. Some patients present primarily with dyspnea, fatigue, or syncope rather than typical angina [27].

(5) Syndromic or inflammatory contexts. When CAE is secondary to vasculitides or connective-tissue disease, systemic features—such as fever, rash, and limited joint mobility—may precede the coronary diagnosis [32, 40].

## 7. Management: Evidence Gaps and Pragmatic Strategies

Because CAE is relatively uncommon and high-quality randomized evidence is lacking, major knowledge gaps persist, and practice remains heterogeneous. Treatment should be individualized according to lesion location and morphology, patient characteristics, and clinical presentation. Broadly, strategies include medical therapy and revascularization (percutaneous or surgical).

### 7.1 Medical Therapy: Antithrombotic Choices and Adjunctive Agents

Given the strong association between CAE and atherosclerosis in adults, intensive lifestyle modification and risk-factor control are foundational and should be emphasized in all patients. The most debated issue is antithrombotic therapy. Marked dilatation in CAE promotes slow flow, stasis, and heightened platelet reactivity, creating a prothrombotic milieu. Observational data, however, are inconsistent. Some series report excess adverse events in ectatic/aneurysmal vessels—e.g., higher mortality in CAA versus angiographic controls [41], a 53.6% MACE rate over ~50 months in a CT-identified CAA cohort [42], and in ACS populations, a >3-fold increase in MACE with CAE, with no events among those who received anticoagulation [43]. By contrast, several retrospective studies did not observe any difference in outcomes related to anticoagulation status [1, 44, 45]. Taken together, these nonrandomized signals support the plausibility of intensified antithrombotic therapy in selected high-risk phenotypes on physiologic and pathophysiologic grounds, while underscoring the uncertainty that surrounds routine anticoagulation.

A systematic review incorporating 5039 patients with CAE found that those who received no treatment had a higher risk of MACE compared to patients on dual antiplatelet therapy (DAPT) or aspirin monotherapy [46]. Furthermore, patients receiving anticoagulation therapy showed a lower incidence of MACE, although this finding did not reach statistical significance [46]. A second systematic review, which included a relatively smaller number of cases, similarly affirmed the role of both antiplatelet and anticoagulation therapies in reducing the incidence of MACE and mortality [47].

Randomized evidence remains sparse. In the exploratory, open-label OVER-TIME trial (n = 62) enrolling ACS patients with culprit-vessel CAE, clopidogrel plus rivaroxaban 15 mg once daily did not significantly reduce the 12-month composite of cardiovascular death, recurrent myocardial infarction (MI), or repeat revascularization compared with aspirin–clopidogrel DAPT; bleeding (bleeding academic research consortium [BARC] 1–5) was similar, whereas recurrent MI was numerically less frequent and fibrin clot lysis time was significantly shorter with clopidogrel–rivaroxaban [48]. These findings suggest a pro-fibrinolytic signal without proven clinical superiority, highlighting the need for adequately powered randomized controlled trials in CAE.

In practice, therapy is individualized by thrombotic and bleeding risk, anatomy, and presentation. A conservative baseline is single antiplatelet therapy for stable, low-risk, non-stented CAE; we escalate to DAPT and/or add oral anticoagulation for heavy thrombus burden, ACS at presentation, slow/no-reflow, distal embolization, or intravascular imaging evidence of laminated thrombus. Where anticoagulation is considered, dosing and combinations should be explicitly distinguished from trial regimens (e.g., low-dose direct oral anticoagulants [DOAC] strategies are not equivalent to the 15 mg rivaroxaban tested in OVER-TIME), and duration should be time-limited with reassessment. In our case, during the index ACS with angiographic thrombus, parenteral anticoagulation was combined with DAPT; given advanced age and bleeding concerns, a low-dose non–vitamin K oral anticoagulant was selected at discharge.

Adjunctive anti-ischemic medications may be useful: calcium-channel blockers can improve coronary flow and treat concomitant vasospasm [49, 50]; in a randomized cohort of 60 patients with isolated CAE, intracoronary diltiazem compared to saline increased TIMI flow, reduced TIMI frame count, and modestly raised myocardial blush grade [51]. β-blockers may relieve ischemia by reducing heart rate and oxygen demand [47], though some authors caution about possible unopposed α-adrenergic vasoconstriction in susceptible patients [52]. Nitrates can further dilate ectatic segments, slow flow, and potentially worsen ischemia; they are generally not recommended for isolated CAE without fixed stenosis [53].

### 7.2 Percutaneous Coronary Intervention: Imaging-Guided Revascularization

Evidence for PCI in asymptomatic CAA is limited; most reports concern outcomes in STEMI/ACS settings [8]. In anatomically suitable patients—e.g., severe ectasia with superimposed thrombus and/or significant focal stenosis—PCI is a viable option, but several technical challenges warrant careful planning.

Ectatic culprit vessels in ACS often harbor a heavy thrombus burden. Despite thrombus aspiration and glycoprotein IIb/IIIa inhibitors, distal embolization, no-reflow, and reperfusion injury are frequent [54, 55, 56, 57]; CAE has been identified as an independent predictor of no-reflow after primary PCI [58]. For patients with substantial thrombus and high no-reflow risk, a delayed-stenting strategy after intensive antithrombotic therapy—similar to our case—can be considered; however, routine deferral is not supported by current evidence and should be reserved for selected high-risk anatomies [59, 60].

Long-term, culprit-vessel ectasia in STEMI has been linked to higher reinfarction, often attributed to stent thrombosis [8]. Malapposition from undersizing is a key mechanism; acute thrombus can cause underestimation of the true landing-zone diameter [4]. Even in non-ACS settings, stent sizing in ectatic vessels is challenging [61], and inappropriate sizing may predispose to stent migration, particularly in giant CAE [4]. Intravascular imaging (IVUS or optical coherence tomography [OCT]) is therefore strongly advisable to characterize lesion morphology, select stent size and landing zones, and confirm expansion/apposition, offering advantages over angiography alone in ectatic segments [4].

Covered stents are primarily used for saccular aneurysms that do not involve major side branches. Their deployment can be technically demanding due to device stiffness, frequent need for larger guide catheters, and the risk of side-branch occlusion post-implantation. When covered stents are unsuitable because of severe tortuosity, calcification, or concern for side branches, stent-assisted coil embolization—adapted from neurointerventional practice—may be an alternative [4].

In PCI practice, for patients with heavy thrombus burden in ectatic culprit vessels, deferred stenting after intensified antithrombotic therapy and IVUS-guided sizing may mitigate the risks of no-reflow and stent malapposition.

### 7.3 Surgical Options for Complex or Giant Lesions

Surgical indications for CAE/CAA are not standardized, and robust comparative data are lacking. Retrospective series suggest no clear MACE difference between surgical and PCI approaches [62, 63]. Surgery is generally favored for left main involvement, multivessel or giant aneurysms (>20 mm or >4× the reference diameter), or in the presence of acute mechanical complications. Operative strategies include coronary artery bypass grafting (CABG), aneurysmectomy, and aneurysm exclusion/plication techniques [4].

## 8. Prognosis

Prognosis in CAE patients is still controversial. Although patients with focal CAE generally have a good prognosis, those with diffuse CAE are at higher risk for MACE [28]. On the other hand, in acute myocardial infarction, a meta-analysis of 7 observational studies (13,499 patients in total) did not show any difference in all-cause mortality or in MACE between CAE patients and those without CAE [64]. This suggests that all CAE patients require long-term monitoring and close follow-up, with treatment strategies tailored according to the extent of lesion involvement and concomitant diseases.

## 9. Synthesis and Clinical Implications

This case of inferior STEMI combined with RCA ectasia intuitively demonstrates the main pathophysiology repeatedly mentioned in the literature: slow/turbulent blood flow within the ectatic segment promotes *in situ* thrombosis, distal embolization, and increases the risk of coronary no-reflow. Our staged strategy—first achieving medical stabilization and restoring TIMI 3 flow, followed by IVUS-guided stent sizing and landing zone selection before implantation—did yield a favorable outcome and fits into the current ACS/PCI practice framework. However, no standardized pathway exists for such procedures; we define this as an operator-selected strategy, and the certainty in the evidence is currently low.

The CAE–CAA phenotype has practical significance and influences treatment decision-making. Intravascular imaging guidance plays an important role in PCI, whereas surgical treatment is typically reserved for the left main, giant, or complex aneurysms. Antithrombotic therapy remains controversial, with the choice and dosage of agents playing a decisive role in long-term management. In CAE-associated STEMI, adding an oral anticoagulant to antiplatelet therapy currently represents a reasonable option, although no major society guidelines have yet provided specific recommendations.

## 10. Limitations

Limitations exist in the current evidence base. First, there is heterogeneity in the definitions of CAE/CAA. Second, analyses of antithrombotic therapy may be subject to confounding by indication and time-dependent biases. Finally, interventional treatments, particularly stent implantation, are affected by operator and center effects as well as small sample sizes and non-standardized outcome definitions. In the absence of clear guidelines where a certainty of evidence level is assigned to key conclusions, we stress individualized therapy depending on the specific clinical context of the patient.

## 11. Conclusions

CAE is a clinically challenging condition characterized by the diffuse or focal dilatation of epicardial arteries. Patients with CAE who present with ACS require appropriate decision-making, given the potentially life-threatening nature of this condition. This case presentation emphasizes an important concept: in STEMI, where the ultimate goal is restoration of TIMI 3 flow, the nature of the culprit vessel is irrelevant. Intravascular imaging may provide vital guidance during PCI, especially stenting, and is often decisive for both immediate procedural success and long-term prognosis. Comprehensive medical management, especially rational antithrombotic therapy, may bring substantial benefit.

Despite major advances in interventional technologies and pharmacotherapies, the best overall treatment strategy remains controversial because of a paucity of large, well-designed randomized controlled trials. In the absence of these studies, current therapeutic decisions should be guided by operator and clinician experience. Management needs to be tailored to specific CAE location and morphology, unique patient features, and overall clinical presentation.

## Abbreviations

CAE, Coronary artery ectasia; STEMI, ST-segment elevation myocardial infarction; ACS, acute coronary syndromes; RCA, right coronary artery; LAD, left anterior descending; LCx, left circumflex; PDA, posterior descending artery; TIMI, Thrombolysis in Myocardial Infarction; IVUS, intravascular ultrasound; CAA, coronary artery aneurysm; MACE, major adverse cardiovascular events; MMPs, matrix metalloproteinases; DAPT, dual antiplatelet therapy; BARC, bleeding academic research consortium; DOAC, direct oral anticoagulants; OCT, optical coherence tomography; CABG, coronary artery bypass grafting.
